# The relationship between epicuticular long-chained hydrocarbons and surface area - volume ratios in insects (Diptera, Hymenoptera, Lepidoptera)

**DOI:** 10.1371/journal.pone.0175001

**Published:** 2017-04-06

**Authors:** Adrian Brückner, Michael Heethoff, Nico Blüthgen

**Affiliations:** Ecological Networks, Department of Biology, Technische Universität Darmstadt, Darmstadt, Germany; Universidade de Sao Paulo Faculdade de Filosofia Ciencias e Letras de Ribeirao Preto, BRAZIL

## Abstract

Long-chain cuticular hydrocarbons (CHCs) are common components of the epicuticle of terrestrial arthropods. CHC serve as a protective barrier against environmental influences but also act as semiochemicals in animal communication. Regarding the latter aspect, species- or intra-functional group specific CHCs composition and variation are relatively well studied. However, comparative knowledge about the relationship of CHC quantity and their relation to surface area—volume ratios in the context of water loss and protection is fragmentary. Hence, we aim to study the taxon-specific relationship of the CHC amount and surface-area to volume ratio related to their functional role (e.g. in water loss). We focused on flower visiting insects and analyzed the CHC amounts of three insect orders (Hymenoptera, Lepidoptera and Diptera) using gas chromatography—mass spectrometry (GC-MS). We included 113 species from two grassland plots, quantified their CHCs, and measured their body mass and surface area. We found differences in the surface area, CHCs per body mass and the CHC density (= amount of CHCs per surface area) across the three insect taxa. Especially the Hymenoptera had a higher CHC density compared to Diptera and Lepidoptera. CHC density could be explained by surface area-volume ratios in Hymenoptera but not in Diptera and Lepidoptera. Unexpectedly, CHC density decreased with increasing surface area—volume ratios.

## Introduction

Epicuticular long-chain hydrocarbons (cuticular hydrocarbons = CHCs) form the outermost barrier of many terrestrial insects and are relevant for the interactions between animals and their environment, e.g. as waterproofing agents against water loss [1–3] or in chemical communication [4]. In this context, cuticular hydrocarbons serve e.g. as sex/gender recognition pheromones, kairomones, species recognition cues, interspecies recognition cues or as cues for chemical mimicry [5–9]. Hence, CHCs are effected by selection arising from both functions [10]. Furthermore, hydrocarbons are also present on the cuticle of several other terrestrial arthropods, presumably having similar functions in communication and waterproofing [11, 12]. The function of CHCs as a protective barrier against water loss has been hypothesized, but only partly experimentally proven [2, 13–15]. These predictions based on the fact that long-chain hydrocarbons are extremely hydrophobic and do not interact with water [16]. Thus CHCs are thought to be a preadaptation of arthropods to invade terrestrial ecosystems [17]. The epicuticular layer is composed of wax esters, fatty acids, sterols, long-chain alcohols and the actual CHCs which are hydrocarbons with a chain length of >21 carbon atoms in the molecular backbone [4, 18, 19]. The composition is characteristic for different functions of CHCs in protective as well as communicative contexts. In chemical communication, even small compositional differences can cause an entire change of the message and the behavioral response [4]. Regarding CHCs as protective barriers against water loss, a compositional change can lead to different physical properties of the surface lipids, which alters ecophysiological responses [13, 14, 20, 21]. Furthermore, there is growing evidence that different water loss rates in arthropods could be explained by the amount of CHCs [21–24]. Most of these studies, however, only focused on model species rather than providing comparative studies across species and taxa. In comparative context, three different mechanisms may explain the relationship of CHCs and water loss; the total amount of CHCs, the CHC composition (i.e. the proportion of alkanes, branched alkanes and alkenes), or a combination of both [16, 20–23].

Here we focus on the amount of CHCs of insects to explain the relationship of CHCs and water loss, but also include other general structural parameters as body size (using the surface area, the surface area—volume ratio and the shape), which influences this relationship [25]. We used a diverse community of flower visitors (including the three orders Diptera, Hymenoptera and Lepidoptera) as a model system to comparatively investigate their surface areas, surface area—volume ratios and the CHC amounts. We specifically ask whether there is a taxon-specific relationship of CHC amount and surface-area to volume ratio with a potential effect on water loss. Since smaller insects with higher surface area—volume ratios loose more water compared to larger species with lower surface area—volume ratios [25], we hypothesize that these smaller insects should generally have a higher CHC density (= amount of CHCs per surface area) to avoid desiccation.

## Materials and methods

### Insect sampling

Animals were collected from May to September 2013 on two grassland sites around Darmstadt (Hesse) in southwest Germany. The main study site “Streit-Gewann” (Griesheim: 49°50'43''N, 8°33'57''E) was a sandy grassland plot surrounded by crop fields and inland dunes [26]. Additionally, specimens were collected on a meadow on the “Campus Lichtwiese” (Darmstadt: 49°51'44''N, 8°'40'36''E). Insects sitting on flowers were caught with a net and directly transferred into vials sealed with foam material. Insects were killed in the freezer at –18°C and stored there for further analyses. Since all insects were collected sitting/foraging on flowers we refer to them as “flower visitors” in this study. No protected areas were involved; permission for insect sampling was issued by regional authorities (UNB Landkreis Darmstadt-Dieburg).

### Cuticular hydrocarbon analyses

Cuticular hydrocarbons (CHCs) of 113 species (n = 375 individuals) were chemically analyzed and their CHC amounts were quantified. We extracted CHCs from the body surface of the insects by submersing the specimens in n-hexane (98% purity for gas-chromatography, Fam. Merck, Germany) at 17°C for 10 minutes. After this time hexane extracts were soaked up with a glass pipette, transferred to a new GC glass vial and the solvent was evaporated under gentle nitrogen gas flow until dryness. Depending on the insect size to adjust the concentration, residuals were subsequently dissolved in 100 to 3000 μl n-hexane (details see S1 Data) containing hexadecane (β = 15 ng/μl; ≥99.8%, analytical standard, purchased from Sigma-Aldrich, Germany) as an internal standard. We analyzed the samples with a QP 2010ultra GC-MS (Shimadzu, Japan). The gas chromatograph (GC) was equipped with a ZB-5MS fused silica capillary column (30 m x 0.25 mm ID, df = 0.25 μm) from Phenomenex (USA). 1 μl sample aliquots were injected by using an AOC-20i autosampler-system from Shimadzu into a split/splitless-injector, which operated in splitless-mode. Injection temperature and pressure were set to 310°C and 250 kPa, respectively, sampling-time was 2 minutes. Hydrogen was used as carrier-gas with a constant flow rate of 1.1 ml/min. The temperature of the GC oven was raised from initial 60°C for 1 min, to 250°C with a heating-rate of 20°C/min, to 320°C with a heating-rate of 5°C/min and then an isothermal hold at 320°C for 5 min. Electron ionization mass spectra were recorded at 70 eV with a scan rate of 2 scans/sec from m/z 40 to 650. The ion source of the mass spectrometer and the transfer line were kept at 280°C and 325°C, respectively.

### Surface measurements

After the n-hexane extraction all specimens were identified to species level, if possible. Subsequently we dried the insects until weight constancy for at least 4 days at 60°C and determined their dry weight using a microbalance (Mettler Toledo, XS3DU, readability 0.1 μg and 1 μg repeatability). The surface areas were calculated using Eq 1; values with index L are taken from (see [25] and S1 Data) and values with index E are measured in this study; based on the specimens´ dry weight. Extremely hairy species (e.g. species of the genus *Bombus*) were shaved with a soldering-iron. We used literature data of the same species or from the same genus, if we were not able to determine the individuals to species level. A modified structured light scanner as described in [25] was used for 3D imaging. Briefly: a beamer and a camera were installed on a stand with fixed positions. The mounted insects were fixed in a tap holder on a step-motor. 24 scans were taken from each insect (15° rotation angle for each scan) and were fused to generate a point cloud which was finally meshed to a 3D-surface. Surface areas and volumes were measured in amira 4.0.1 (FEI Visualization Sciences Group, USA), and all the measures are listed in the S1 Data.

surface area=surface areaLdry weightL23x  dry weightE23 (1)

### Data processing and statistical analysis

Shimadzu GC-MS Postrun Analysis (v. 2.71) was used for the evaluation of chromatographic data. We included all unpolar, acyclic cuticular hydrocarbons (i.e. alkenes, methyl-branched alkanes, n-alkanes) with proportions > 1% of the total blend and chain lengths >C21 for our analyses, since only these compounds could be identified and are considered as CHCs [27]. We analyzed the bulk CHCs per individual, i.e. we check all peaks in all chromatograms and only integrated those with n-alkane/branched-alkane or alkene diagnostic ions (*m/z* = 43, 57, 71, 85, 99 or *m/z* = 41, 55, 69, 83, 97, respectively). In the range of relevant concentrations, the responses of the alkane standard relative to hexadecane were determined by a dilution and turned out to be close to 1, allowing an approximation of CHCs in ng as given in Eq 2 (with β as mass concentration of the internal standard, V_s_ as total sample volume, DW as dry weight and SA as surface area). The absolute amount of CHCs was divided by the dry weight (mg) or the surface area (mm^2^) to describe the relative amount of CHCs per body mass (ng/mg) and the CHC density (ng/mm^2^), respectively.

$$CHC\;per\;DW\;or\;SA\;\;=\;\;\frac{\beta \left(internal\;standard\right)*\;\sum area\;\left(CHC\right)*Vs}{area\;\left(internal\;standard\right)}/\;DW\;\text{or}\;SA$$ (2)

We statistically analyzed the CHC amount per dry weight and per surface area using ANOVA and TukeyHSD [28] post-hoc test. We checked for normal distribution of residuals and the homogeneity of variance and transformed the data where necessary (CHCs per dry weight was square root-transformed; CHCs per surface area was log-transformed). Furthermore, we used simple linear models and ANOVA to analyze the relationship of the CHC amount per surface area, the relationship of the insect order and the surface area-volume ratio, as well as the relationship of the surface area, the insect order and body mass. The dependent variables were square root-transformed (CHCs per surface area) or log-transformed (surface area) to meet constant error variance and normality of the residuals. We used the mean values of the species for all analyses to avoid pseudoreplication, so the different species (n = 113) were considered as replicates. All statistics were performed with R [29].

## Results

Overall, we collected 375 specimens representing 113 flower visiting insect species of three different orders, namely, Diptera (n = 54 species and 136 specimen), Hymenoptera (n = 33 species and 159 specimen) and Lepidoptera (n = 26 species and 80 specimen).

**Fig 1:**
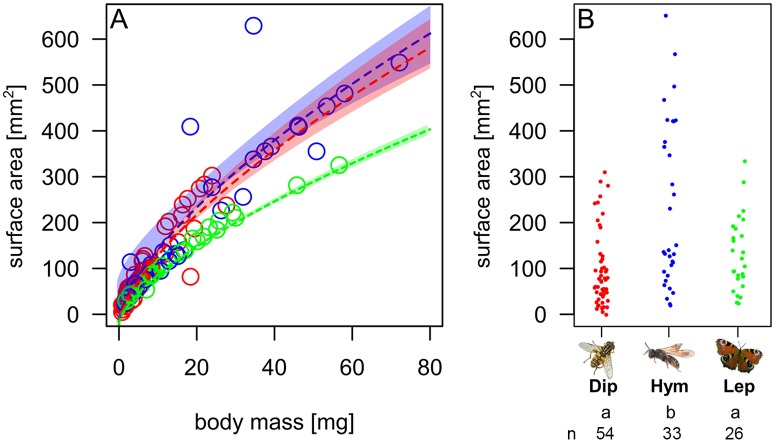
Surface areas of three different orders of flower-visiting insects. (A) Body mass prediction of surface area in three insect orders Diptera, Hymenoptera and Lepidoptera. Colored transparent areas represent 95% confidence intervals. (B) Surface area across the three insect orders (n = 113 species). Letters indicate homogenous groups (TukeyHSD).

The surface area of the insects increased with a 2/3 power term over body mass (overall linear model, two-way ANOVA: F_5,107_ = 124, R^2^ = 0.85, P < 0.001, Table 1, Fig 1A). However, the significant interaction of the linear model (Table 1) revealed an order-specific increase of the surface area with body mass^2/3^. Furthermore, the surface area [mm^2^] varied among the insect orders (One-way ANOVA: F_2,110_ = 13.47; P < 0.001, Fig 1B). Generally, hymenopterans had a larger surface area (mean = 235±168 mm^2^) and higher value-range than dipterans (mean = 100±77 mm^2^) and lepidopterans (mean = 135±76 mm^2^ (Table 2 and Fig 1B).

**Table 1:** How body mass predicts the insects’ surface. The effect of insect order and body mass^2/3^ (mg) on the surface area (mm^2^) across 113 species of insects. The surface area was log-transformed before the analysis. Significant predictors (P < 0.05) in bold.

	df	F	P
order	2	63.16	**< 0.001**
body mass^2/3^ (mg)	1	455.26	**< 0.001**
order x body mass^2/3^ (mg)	2	19.25	**< 0.001**
residuals	107		

**Table 2:** Surface area and cuticular hydrocarbon characteristics of insects. Results of pairwise comparisons among the different insect orders. Significant comparisons (TukeyHSD, P < 0.05) in bold. Hym = Hymenoptera, Dip = Diptera, Lep = Lepidoptera.

	Hym-Dip	Hym-Lep	Dip-Lep
I—surface area (mm^2^)	**< 0.001**	**0.02**	0.18
II—CHC/DW (ng/mg)	**0.043**	**< 0.001**	**< 0.001**
III—CHC/SA (ng/mm^2^)	**< 0.001**	**< 0.001**	0.33

**Fig 2:**
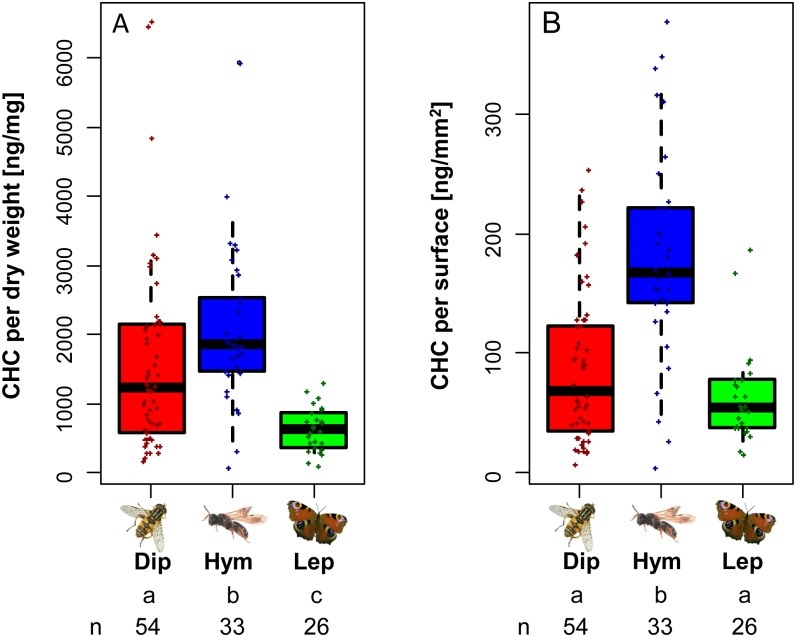
Quantitative variation of CHCs among flower visitors. Cuticular hydrocarbons per dry weight (A) and per surface area (B), across three insect orders (n = 113 species). Letters indicate homogenous groups (TukeyHSD).

The amount of CHCs, standardized by the insect’s dry weight (ng/mg), varied among the flower-visiting insect orders (One-way ANOVA: F_2,110_ = 15.99; P < 0.001, Fig 2A). The mean amount of CHCs in Hymenoptera (2074±1098 ng/mg) was about 1.5 times higher than in Diptera (1590±1380 ng/mg) and even three times higher than Lepidoptera (634±311 ng/mg) (Table 2 and Fig 2A). The CHC density (ng/mm^2^) was also significantly different among the three orders (One-way ANOVA: F_2,110_ = 27.40; P < 0.001, Fig 2B). Again, the CHC density in hymenopterans (181±87 ng/mm^2^) was more than twice that of dipterans (87±62 ng/ mm^2^) and about three times higher than in lepidopterans (64±39 ng/ mm^2^), whereas the densities in the latter two were not significantly different from each other (Table 2 and Fig 2B).

**Fig 3:**
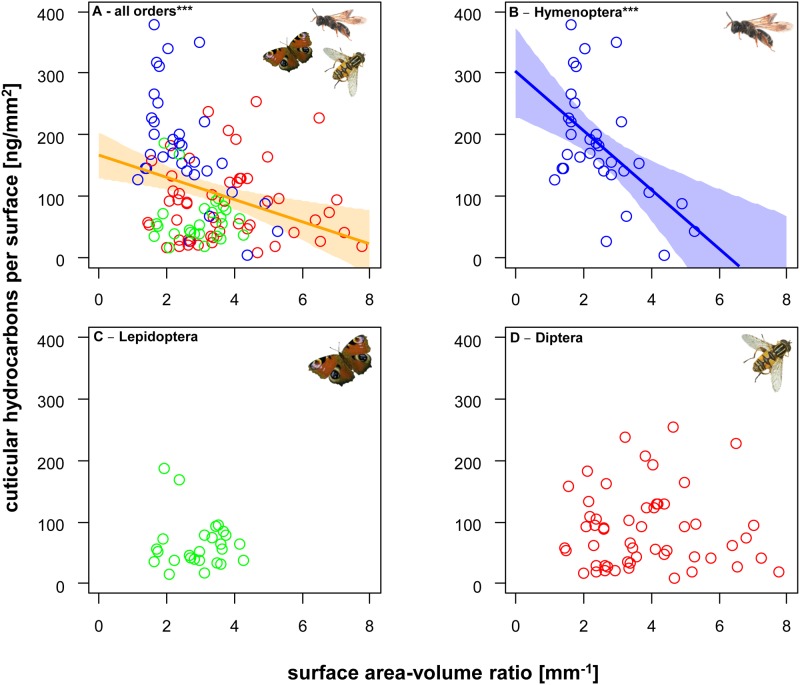
The relationship between surface-area volume ratios and CHC density. Surface area-volume ratio predictions of cuticular hydrocarbon density of all flower visitor species (A), Hymenoptera (B), Lepidoptera (C) and Diptera (D). Colored transparent areas represent 95% confidence intervals. *P < 0.05, **P < 0.01, ***P < 0.001.

The CHC density per surface area (ng/mm^2^) of the different flower visitors significantly decreased with their surface area-volume rate (mm^-1^) (overall linear model, two-way ANOVA: F_5,107_ = 14.4, R^2^ = 0.40, P < 0.001, Table 3, Fig 3A). However, the significant interaction of the linear model (Table 3) revealed an order-specific decrease of CHC density with increasing surface area-volume ratio. This relationship was only significant for Hymenoptera (F_1,31_ = 13.2, R^2^ = 0.30, P < 0.001; Fig 3B), but not for Lepidoptera (F_1,24_ = 0.2, R^2^< 0.01, P = 0.63; Fig 3C) or Diptera (F_1,52_< 0.1, R^2^< 0.01, P = 0.88; Fig 3D). The same trends were also found for the relationship of CHC density per surface area (ng/mm^2^) and body mass (mg) for the three orders (see S1 File): Hymenoptera (F_1,31_ = 7.6, R^2^ = 0.18, P < 0.001), Lepidoptera (F_1,24_ = 2.7, R^2^ = 0.10, P = 0.11) and Diptera (F_1,52_ = 1.1, R^2^< 0.01, P = 0.30).

**Table 3:** How surface-area volume ratio predicts the CHC density of flower visitors. The effect of insect order and surface area-volume ratio (mm^-1^) on the cuticular hydrocarbon surface density (ng/ mm^2^). CHC density was sqrt-transformed before analysis. Significant predictors (P < 0.05) in bold.

	df	F	P
order	2	27.17	**< 0.001**
surface/volume (m^-1^)	1	4.46	**0.037**
order x surface/volume (m^-1^)	2	6.58	**0.002**
residuals	107		

## Discussion

Cuticular hydrocarbons (CHCs) of the three insect taxa differ in their amounts per body mass and surface area (Fig 2, Table 2). Furthermore, the CHC amount per surface area (= CHC density) can be explained with the insects’ surface area-volume ratio in Hymenoptera, but not in Diptera and Lepidoptera (Fig 3, Table 3). Such CHCs are potentially relevant functional species traits—here represented by active, exposed foraging adults of flower visiting insects—which shape the animals’ responses to environmental conditions such as thermal resilience, alternated phenology, land-use and disturbance or elevation [30–35].

The surface area of any object increases isometrically (with power 2/3) with its body volume or mass (Fig 1A), and allometrically with variable shape. The surface of the cuticle is functionally important for interactions of animals with their environment and may therefore be crucial for the animal’s ecophysiological responses [25, 36, 37]. Some studies [8, 38] thus used geometric approximations of the insects’ surfaces. Here we used direct surface measurements of the flower visitors (Fig 1B) using a modified structured light 3D scanner [25] and were therefore able to calculate the CHC density per surface area [ng/mm^2^].

The CHC density per body mass or per surface area was highest in Hymenoptera (Fig 2), an insect order which is known for their high degree of sociality and therefore frequent communication via cuticular compounds [39, 40], while Diptera and Lepidoptera seem to invest less resources (per body mass) to build up their CHCs. This difference between orders was even more pronounced in the CHC density (compare Fig 2A and 2B). For some Hymenoptera, namely bumblebees (Apidae, *Bombus*), the CHC density is particularly high, and substantial amounts are left as chemical CHCs footprints on flowers after each visit [41]. The higher density of CHCs in some dipterans compared to lepidopterans (not significant given the high variance of CHC density among dipterans, Fig 2B) may derive from the importance of such hydrocarbons as abundant sex pheromones in some flies [42], whereas lepidopterans use polyunsaturated hydrocarbons and other compound classes for chemical signaling [43, 44]. From a methodological point of view, our results (Fig 2A and 2B; Table 2) demonstrate the difference between simple body mass standardization versus the use of actual surface area to calculate the CHC densities. The fact that insects of different orders have different shapes, e.g. sphericity [25] strongly contribute to these differences. Hence, the commonly used body mass standardization (see e.g., [37, 45–47] for some examples in ecology; see also [48] for one of the few examples in chemical ecology) may not be a suitable approximation. A standardization by body mass (dry weight) may be appropriate to describe the relative resource allocation of an insect to its chemical substances on the cuticle, but it may not be ideal to allow conclusions about the importance of surface area. Thus, we suggest that the CHC density per surface area could be included as functional trait in future studies, particularly for environmental responses such as thermal resilience, but maybe also to study the degree of social integration in mimicry-systems such as acquired chemical mimicry in myrmecophiles and their host ants [8, 38].

Since smaller insects have a higher surface area—volume ratio (see also Fig 1), predicted by the geometric square-cube law [49] and confirmed by surface measurements on insects [25], smaller species typically lose more water than larger ones. Hence, to protect the insect from water loss, we expected to find a higher CHC density per surface area [ng/mm^2^] in smaller insects. Surprisingly, we found the opposite trend for Hymenoptera and no trend for Lepidoptera and Diptera (Fig 3). The huge variation (and low *R*^2^ values of the CHC density relationship with surface area—volume ratio) indicates that species are highly idiosyncratic even within each taxon. This finding is inconsistent with the experimental evidence for a quantitative effect of CHCs on water loss, which were however based on experiments with a few model species only [21, 22]. Thus, CHC amount/density does not seem to be a general, singular predictor of physical properties of the epicuticle across species and taxa (see also [15]). Compositional aspects of CHC in insects which have been intensively studied focusing on general ecology and evolution [39, 50–53] seem to be crucial, and overall compositional differences in CHC may influence the insects’ ecophysiological responses like water loss [4, 20, 21]. Recently, Menzel et al. [54] showed that the evolutionary patterns of CHC composition on a global scale are influenced by regional climatic conditions. However, the precise contribution of CHC composition to water loss prevention is not fully understood. There is some evidence that composition (e.g. higher proportion of alkenes) influences the water permeability of insect cuticles (reviewed by [2, 15]; see also [54]), but the predictions of water loss based on a multicomponent system like insect epicuticular hydrocarbons remain uncertain [13, 14], and most probably a taxon-specific combination of CHC quality and quantity may be required to allow a better prediction of variation in water loss in insects.

## Supporting information

S1 File How CHC per surface area are predicted by order and body mass. (DOCX)

S1 Data The file summarizes all the relevant data that have been used in the statistical analyses. (PDF)

## References

[1] Gibbs AG. Water balance in desert *Drosophila*: lessons from non-charismatic microfauna. Comp Biochem Physiol A Mol Integr Physiol. 2002;133(3):781–9. 12443934 10.1016/s1095-6433(02)00208-8

[2] Gibbs AG. Thermodynamics of cuticular transpiration. J Insect Physiol. 2011;57(8):1066–9. 10.1016/j.jinsphys.2011.05.003 21605563

[3] Hadley NF. Water relations of terrestrial arthropods. London: Academic Press; 1994.

[4] Blomquist GJ, Bagnères A-G. Insect hydrocarbons. Cambridge Cambridge University Press; 2010.

[5] Colazza S, Aquila G, De Pasquale C, Peri E, Millar JG. The egg parasitoid *Trissolcus basalis* uses n-nonadecane, a cuticular hydrocarbon from its stink bug host *Nezara viridula*, to discriminate between female and male hosts. J Chem Ecol. 2007;33(7):1405–20. 10.1007/s10886-007-9300-7 17508134

[6] Jennings JH, Etges WJ, Schmitt T, Hoikkala A. Cuticular hydrocarbons of *Drosophila montana*: geographic variation, sexual dimorphism and potential roles as pheromones. J Insect Physiol. 2014;61:16–24. 10.1016/j.jinsphys.2013.12.004 24373710

[7] Sharma KR, Enzmann BL, Schmidt Y, Moore D, Jones GR, Parker J, et al Cuticular hydrocarbon pheromones for social behavior and their coding in the ant antenna. Cell Rep. 2015;12(8):1261–71. 10.1016/j.celrep.2015.07.031 26279569

[8] von Beeren C, Schulz S, Hashim R, Witte V. Acquisition of chemical recognition cues facilitates integration into ant societies. BMC Ecol. 2011;11:30 10.1186/1472-6785-11-30 22133503 PMC3271039

[9] Wurdack M, Herbertz S, Dowling D, Kroiss J, Strohm E, Baur H, et al Striking cuticular hydrocarbon dimorphism in the mason wasp *Odynerus spinipes* and its possible evolutionary cause (Hymenoptera: Chrysididae, Vespidae). Proc Biol Sci. 2015;282(1821):20151777 10.1098/rspb.2015.1777 26674944 PMC4707744

[10] Chung H, Carroll SB. Wax, sex and the origin of species: Dual roles of insect cuticular hydrocarbons in adaptation and mating. Bioessays. 2015;37(7):822–30. 10.1002/bies.201500014 25988392 PMC4683673

[11] Raspotnig G, Krisper G, Fauler G, Leis HJ. Distinctive cuticular hydrocarbon profiles in oribatid mites (Acari: Oribatida). Ann Zool (Warszawa). 2008;58(2):445–52.

[12] Wachter GA, Muster C, Arthofer W, Raspotnig G, Fottinger P, Komposch C, et al Taking the discovery approach in integrative taxonomy: decrypting a complex of narrow-endemic Alpine harvestmen (Opiliones: Phalangiidae: Megabunus). Mol Ecol. 2015;24(4):863–89. 10.1111/mec.13077 25583278

[13] Gibbs AG. Water-proofing properties of cuticular lipids. Am Zool. 1998;38:471–82.

[14] Gibbs AG. Lipid melting and cuticular permeability: new insights into an old problem. Journal of Insect Physiology. 2002;48:391–400. 12770088 10.1016/s0022-1910(02)00059-8

[15] Gibbs AG, Rajpurohit S. Cuticular lipid and water balance In: Blomquist GJ, Bagneres AG, editors. Insect hydrocarbons. Cambridge: Cambridge University Press; 2010 p. 100–21.

[16] Hadley NF. Lipid water barriers in biological systems. Prog Lipid Res. 1989;28(1):1–33. 2682668 10.1016/0163-7827(89)90005-2

[17] Gibbs AG, Pomonis JG. Physical-properties of insect cuticular hydrocarbons—the effects of chain-length, methyl-branching and unsaturation. Comp Biochem Physiol B Comp Biochem. 1995;112:391–400

[18] Blomquist GJ, Nelson DR, Derenobales M. Chemistry, biochemistry, and physiology of insect cuticular lipids. Arch Insect Biochem Physiol. 1987;6:227–65.

[19] Gibbs AG, Markow TA. Effects of age on water balance in *Drosophila* species. Physiol Biochem Zool. 2001;74(4):520–30. 10.1086/322162 11436136

[20] Stinziano JR, Sove RJ, Rundle HD, Sinclair BJ. Rapid desiccation hardening changes the cuticular hydrocarbon profile of *Drosophila melanogaster*. Comp Biochem Physiol A Mol Integr Physiol. 2015;180:38–42. 10.1016/j.cbpa.2014.11.004 25460832

[21] Gefen E, Talal S, Brendzel O, Dror A, Fishman A. Variation in quantity and composition of cuticular hydrocarbons in the scorpion *Buthus occitanus* (Buthidae) in response to acute exposure to desiccation stress. Comp Biochem Physiol A Mol Integr Physiol. 2015;182:58–63. 10.1016/j.cbpa.2014.12.004 25499238

[22] Arcaz AC, Huestis DL, Dao A, Yaro AS, Diallo M, Andersen J, et al Desiccation tolerance in *Anopheles coluzzii*: the effects of spiracle size and cuticular hydrocarbons. J Exp Biol. 2016;219(Pt 11):1675–88. 10.1242/jeb.135665 27207644 PMC4920233

[23] Hadley NF. Epicuticular lipids of desert tenebrionid beetle, *Eleodes armata*—seasonal and acclimatory effects on composition. Insect Biochem. 1977;7:277–83.

[24] Toolson EC. Effects of rearing temperature on cuticle permeability and epicuticular lipid-composition in *Drosophila pseudoobscura*. J Exp Zool. 1982;222:249–53.

[25] Kühsel S, Brückner A, Schmelzle S, Heethoff M, Blüthgen N. Surface area—volume ratios in insects. Insect Science. 2016. 10.1111/1744-7917.12362 27234132

[26] Freund L, Eichberg C, Retta I, Schwabe A. Seed addition via epizoochorous dispersal in restoration: an experimental approach mimicking the colonization of bare soil patches. Appl Veg Sci. 2014;17(1):74–85.

[27] Nelson DR, Blomquist GJ. Insect waxes. Hamilton RJ, editor. Dundee: The Oily Press; 1995.

[28] Tukey JW. Comparing individual means in the analysis of variance. Biometrics. 1949;5(2):99–114. 18151955

[29] Team RDC. R: A language and environment for statistical computing. R Foundation for Statistical Computing, Vienna, Austria ISBN 3-900051-07-0, URL http://www.R-project.org. 2015.

[30] Blüthgen N, Klein AM. Functional complementarity and specialisation: The role of biodiversity in plant-pollinator interactions. Basic Appl Ecol. 2011;12(4):282–91.

[31] Cariveau DP, Williams NM, Benjamin FE, Winfree R. Response diversity to land use occurs but does not consistently stabilise ecosystem services provided by native pollinators. Ecol Lett. 2013;16(7):903–11. 10.1111/ele.12126 23692675

[32] Fründ J, Dormann CF, Holzschuh A, Tscharntke T. Bee diversity effects on pollination depend on functional complementarity and niche shifts. Ecology. 2013;94(9):2042–54. 24279275 10.1890/12-1620.1

[33] Kühsel S, Blüthgen N. High diversity stabilizes the thermal resilience of pollinator communities in intensively managed grasslands. Nat Commun. 2015;6:7989 10.1038/ncomms8989 26258282 PMC4918356

[34] Mori AS, Furukawa T, Sasaki T. Response diversity determines the resilience of ecosystems to environmental change. Biol Rev. 2013;88(2):349–64. 10.1111/brv.12004 23217173

[35] Peters MK, Peisker J, Steffan-Dewenter I, Hoiss B. Morphometric traits mediating cold performance in bees and their distribution along elevational gradients. J. Biogeogr. 2016.

[36] Lighton JRB, Feener DH. Water-loss rate and cuticular permeability in foragers of the desert ant *Pogonomyrmex rugosus*. Physiol Zool. 1989;62(6):1232–56.

[37] Porter WP, Gates DM. Thermodynamic equilibria of animals with environment. Ecol Monogr. 1969;39(3):227–&.

[38] Nehring V, Dani FR, Calamai L, Turillazzi S, Bohn H, Klass KD, et al Chemical disguise of myrmecophilous cockroaches and its implications for understanding nestmate recognition mechanisms in leaf-cutting ants. BMC Ecol. 2016;16:35 10.1186/s12898-016-0089-5 27495227 PMC4974750

[39] Leonhardt SD, Menzel F, Nehring V, Schmitt T. Ecology and evolution of Communication in social insects. Cell. 2016;164(6):1277–87. 10.1016/j.cell.2016.01.035 26967293

[40] Liebig J. Hydrocarbon profiles indicate fertility and dominance status in ant, bee, and wasp colonies In: Blomquist GJ, Bagneres AG, editors. Insect hydrocarbons. Cambridge: Cambridge University Press; 2010 p. 254–82.

[41] Witjes S, Eltz T. Hydrocarbon footprints as a record of bumblebee flower visitation. J Chem Ecol. 2009;35(11):1320–5. 10.1007/s10886-009-9720-7 20013038

[42] Ferveur J-F, Cobb M. Behavioral and evolutionary roles of cuticular hydrocarbons in Diptera In: Blomquist GJ, Bagneres AG, editors. Insect hydrocarbons. Cambridge: Cambridge University Press; 2010.

[43] Millar J. Polyene hydrocarbons, epoxides, and related compounds as components of lepidopteran pheromone blends In: Blomquist GJ, Bagneres AG, editors. Insect hydrocarbons. Cambridge: Cambride University Press; 2010 p. 390–448.

[44] Millar JG. Polyene hydrocarbons and epoxides: a second major class of lepidopteran sex attractant pheromones. Annu Rev Entomol. 2000;45:575–604. 10.1146/annurev.ento.45.1.575 10761590

[45] Hickman JC, Pitelka LF. Dry weight indicates energy allocation in ecological strategy analysis of plants. Oecologia. 1975;21(2):117–21. 10.1007/BF00345554 28308242

[46] Rosillo-Calle F, de Groot P, Hemstock SL, Woods J. The biomass assessment handbook. London: Earthscan; 2007.

[47] Saint-Germain M, Buddle CM, Larrivee M, Mercado A, Motchula T, Reichert E, et al Should biomass be considered more frequently as a currency in terrestrial arthropod community analyses? J Appl Ecol. 2007;44(2):330–9.

[48] Hadley NF, Blomquist GJ, Lanham UN. Cuticular hydrocarbons of 4 species of colorado hymenoptera. Insect Biochem. 1981;11(2):173–7.

[49] Galilei G. Discorsi e Dimostrazioni Matematiche Intorno a due nuove Scienze Attinenti la Meccanica e i Movimenti Locali. Leiden: Elsevier; 1638.

[50] Howard RW, Blomquist GJ. Ecological, behavioral, and biochemical aspects of insect hydrocarbons. Annu Rev Entomol. 2005;50:371–93. 10.1146/annurev.ento.50.071803.130359 15355247

[51] Ingleby FC. Insect cuticular hydrocarbons as dynamic traits in sexual communication. Insects. 2015;6(3):732–42. 10.3390/insects6030732 26463413 PMC4598662

[52] Kather R, Martin SJ. Cuticular hydrocarbon profiles as a taxonomic tool: advantages, limitations and technical aspects. Physiol Entomol. 2012;37(1):25–32.

[53] Martin SJ, Drijfhout FP. Nestmate and task cues are influenced and encoded differently within ant cuticular hydrocarbon profiles. J Chem Ecol. 2009;35(3):368–74. 10.1007/s10886-009-9612-x 19263166

[54] Menzel F, Blaimer BB, Schmitt T. How do cuticular hydrocarbons evolve? Physiological constraints, climatic and biotic selection pressures act on a complex functional trait in insects. Proc R Soc Lond Biol. in press. 10.1098/rspb.2016.1727 PMC5360911 28298343

