# Supplementary material for: The relationship between epicuticular long-chained hydrocarbons and surface area - volume ratios in insects (Diptera, Hymenoptera, Lepidoptera)
Source: PLoS One. 2017 Apr 6;12(4):e0175001. doi: 10.1371/journal.pone.0175001 (PMC5383148; doi:10.1371/journal.pone.0175001)
Supplement: S1 File — (DOCX) [file pone.0175001.s001.docx]

**Supplementary material – Brückner et al. “The relationship between epicuticular long-chained hydrocarbons and surface area volume - ratios in insects (Diptera, Hymenoptera, Lepidoptera)”**


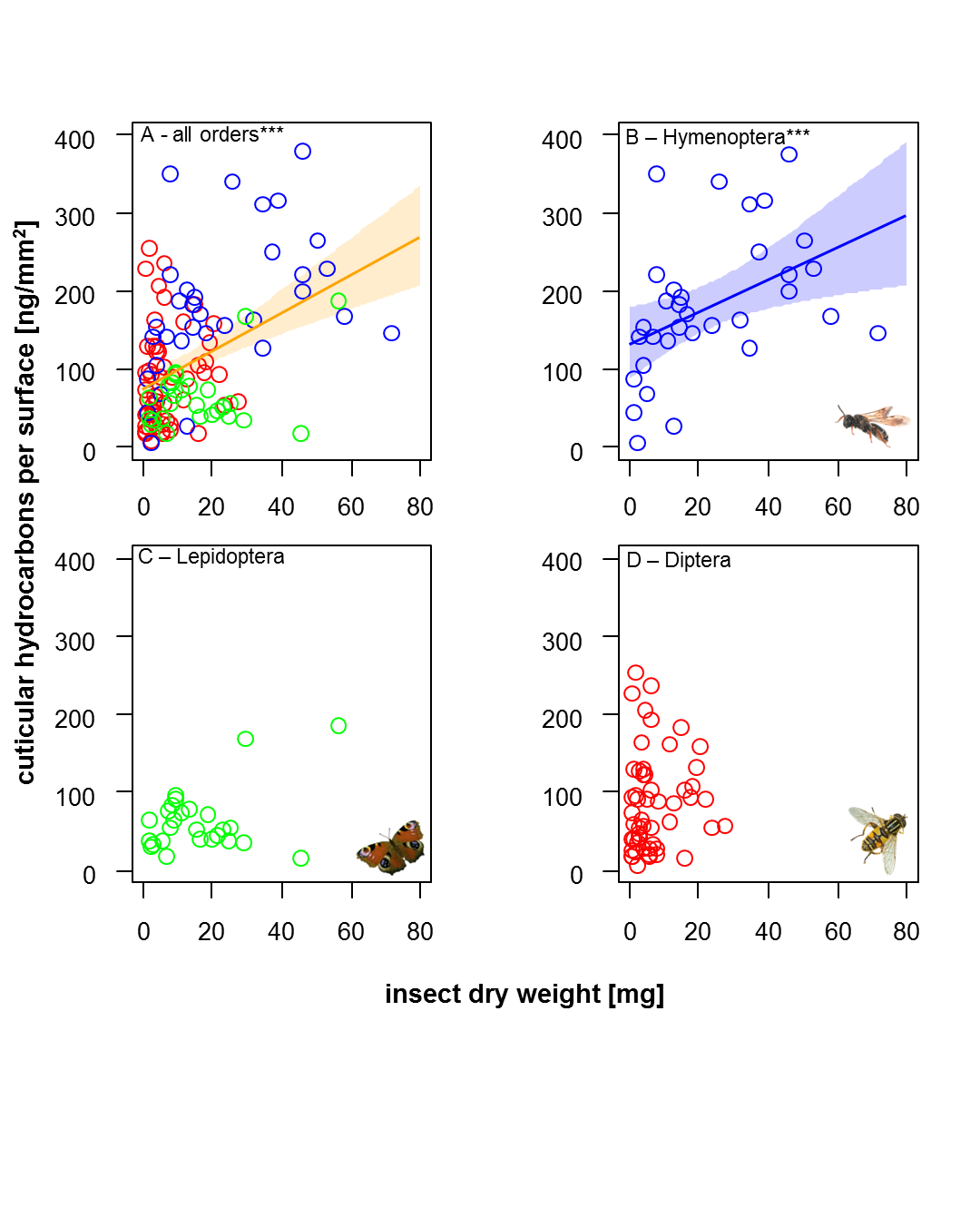


**S1 Fig.** **CHC per surface area and the relation to order and body mass**. How insect dry weight predicts the cuticular hydrocarbon density of all flower visitor species (A), Hymenoptera (B), Lepidoptera (C) and Diptera (D). Colored transparent areas represent 95% confidence intervals. ***p < 0.001

**S1 Text**

The CHC density per surface area [ng/mm^2^] of the different insect orders significantly increased with their body mass [mg] for all orders (linear model, two-way ANOVA: F_5,107_= 12.8, R^2^= 0.37, P < 0.001, Fig. S1 A). This relationship was, however, only significant for hymenoptera (F_1,31_= 7.6, R^2^= 0.18, P = 0.009; Fig. S1 B) and lepidoptera (F_1,24_= 2.7, R^2^= 0.10, P = 0.11; Fig. S1 C), but not for dipterans (F_1,52_= 1.1, R^2^< 0.01, P = 0.30; Fig. S1 D).

**S1 Table. How body mass predicts the CHC density of flower visitors.** The effect of insect order and body mass on the cuticular hydrocarbon surface density [ng/ mm^2^] of flower visitors. CHC density was sqrt-transformed before analysis. Significant predictors (p < 0.05) in bold.

|  | df | F | P |
| --- | --- | --- | --- |
| order | 2 | 25.98 | **< 0.001** |
| body mass [mg] | 1 | 11.58 | **< 0.001** |
| order x body mass [mg] | 2 | 0.28 | 0.75 |
| residuals | 107 |  |  |
