# Supplementary material for: The relationship between epicuticular long-chained hydrocarbons and surface area - volume ratios in insects (Diptera, Hymenoptera, Lepidoptera)
Source: PLoS One. 2017 Apr 6;12(4):e0175001. doi: 10.1371/journal.pone.0175001 (PMC5383148; doi:10.1371/journal.pone.0175001)
Supplement: S1 Data — (PDF) [file pone.0175001.s002.pdf]

ID	Order	Species	surface area in mm^2	CHC in ng	SA/V ratio in mm^-1	dilution in µl	CHC_DW in ng/mg	CHC_SA in ng/mm^2	n
1	Diptera	*Acanthiophilus helianthi*	22.13	896	7.24	100	1281.2	40.5	1
2	Lepidoptera	*Aglais urticae*	285.51	4597	2.07	100	101.1	16.1	1
3	Hymenoptera	*Ambyletes armatorius*	413.17	59951	1.39	100	3306.6	145.1	1
4	Hymenoptera	*Ammophila sabulosa*	633.26	80361	1.12	500	2335.6	126.9	1
5	Hymenoptera	*Andrena* ssp.	98.99	18541	2.36	200	1968.9	187.3	4
6	Hymenoptera	*Anthophora bimaculata*	260.14	42429	1.88	1000	1187.5	163.1	2
7	Lepidoptera	*Apamea monoglypha*	169.2	12182	1.87	100	651	72	1
8	Hymenoptera	*Apis mellifera*	281.08	43624	2.82	500	1664.6	155.2	15
9	Lepidoptera	*Araschnia levana*	126.29	9964	3.11	100	743.8	78.9	1
10	Lepidoptera	*Argynnis aglaja*	329.34	61620	1.93	100	1092.1	187.1	1
11	Lepidoptera	*Autographa gamma*	225.78	7880	1.61	100	270.4	34.9	10
12	Hymenoptera	*Bombus hortorum*	457.45	104024	1.54	1250	1843.4	227.4	3
13	Hymenoptera	*Bombus humilis*	359.5	90342	1.72	1000	2462.2	251.3	6
14	Hymenoptera	*Bombus lapidarius*	412.81	82438	1.61	1500	1812.3	199.7	35
15	Hymenoptera	*Bombus pascuorum*	369.72	117127	1.7	1250	2953.9	316.8	17
16	Hymenoptera	*Bombus pratorum*	415.57	92257	1.62	1250	2037.8	222	4
17	Hymenoptera	*Bombus ruderatus*	414.79	157288	1.62	1500	3327	379.2	2
18	Hymenoptera	*Bombus sylvarum*	341.82	106374	1.78	500	3239.3	311.2	2
19	Hymenoptera	*Bombus sylvestris*	485.38	81544	1.5	1000	1420.8	168	2
20	Hymenoptera	*Bombus terrestris*	552.87	80443	1.38	1500 (10)<br>3000 (7)	1113	145.5	17
21	Diptera	*Botanophila fugax*	34.91	1442	5.76	100	1038.2	41.3	1
22	Diptera	*Chamaesyrphus illustrata*	38.15	2476	3.36	100	719.4	64.9	2
23	Diptera	*Cheilosia gigantea*	82.02	2370	2.35	100	431.8	28.9	1
24	Diptera	*Chloromyia formosa*	63.03	8143	4.17	100	2120.3	129.2	1
25	Lepidoptera	*Chrysaspidia festucae*	207.86	11578	1.68	100	453.9	55.7	1
26	Diptera	*Chrysogaster virescens*	120.08	2209	2.35	100	379.4	18.4	2
27	Diptera	*Chrysotoxum latilimbatum*	161.43	29526	2.12	200	1962.8	182.9	2
28	Hymenoptera	*Coelioxys* cf *elongata*	143.86	24485	2.17	100	1477	170.2	2
29	Lepidoptera	*Coenonympha pamphilus*	84.3	6567	3.74	100	865.8	77.9	7
30	Diptera	*Coenosia tigrina*	79.53	16423	3.82	150	3443.6	206.5	1

31	Lepidoptera	*Colias alfacariensis*	214.65	36061	2.38	200	1175	168	1
32	Hymenoptera	*Colletes marginatus*	131.01	25259	2.19	200	1552.4	192.8	5
33	Diptera	*Cylindromyia auriceps*	67.42	11070	4.96	100	3154.1	164.2	1
34	Diptera	*Cylindromyia brassicaria*	99.13	19152	4.03	150	3122.9	193.2	2
35	Diptera	*Dasysyrphus pinastri*	136.11	8466	2.31	100	727.2	62.2	1
36	Diptera	*Delia platura*	23.44	2220	7.02	100	3047.1	94.7	2
37	Diptera	*Dioctria atricapilla*	99.32	5562	4.1	100	929.6	56	1
38	Diptera	*Dolichopus ungulatus*	58.8	3163	4.44	100	1044.4	53.8	1
39	Lepidoptera	*Dyspessa ulula*	32.56	1221	4.25	100	771.9	37.5	1
40	Diptera	*Empis livida*	86.1	9342	2.17	150	381.6	108.5	3
41	Diptera	*Empis pennispes*	14.69	386	6.53	100	488.4	26.3	1
42	Diptera	*Episyrphus balteatus*	102.73	2959	2.62	150	376.8	28.8	12
43	Diptera	*Eristalis tenax*	241.38	14024	1.42	200	499.1	58.1	3
44	Lepidoptera	*Eublemma minutatum*	45.13	1381	3.6	100	539.2	30.6	3
45	Diptera	*Eupeodes corollae*	84.06	1664	2.94	200	294.3	19.8	1
46	Lepidoptera	*Hadena* sp.	193.85	10022	1.74	100	439.7	51.7	2
47	Hymenoptera	*Halictus* sp.	79.93	11350	3.19	200	1742.8	142	1
48	Diptera	*Helina confinis*	67.1	8213	4.08	100	2261.4	122.4	3
49	Diptera	*Helophilus trivitatus*	191.14	25441	2.14	100	1419.4	133.1	3
50	Diptera	*Hydrotaea meteorica*	27.5	6259	6.49	100	6453.8	227.6	1
51	Hymenoptera	*Hylaeus* sp.	28.45	1238	5.28	100	911	43.5	2
52	Hymenoptera	*Ichneumonidae sp.*	118	16756	2.6	250	5937.6	142	2
53	Hymenoptera	*Lasioglossum albipes*	54.59	8385	3.62	100	2525.3	153.6	19
54	Hymenoptera	*Lasioglossum leucozonium*	90.64	31724	2.98	150	3999.1	350	2
55	Hymenoptera	*Lasioglossum pauxillum*	135.44	24609	2.45	200	1713.7	181.7	2
56	Diptera	*Lonchaea contraria*	41.02	3983	5.29	100	2193.8	97.1	2
57	Diptera	*Lucilia sercata*	101.34	3364	3.38	100	491	33.2	2
58	Lepidoptera	*Lycaena phlaeas*	109.93	8179	3.32	200	741.5	74.4	4
59	Lepidoptera	*Maniola jurtina*	143.25	5658	2.82	100	333.8	39.5	4
60	Hymenoptera	*Megachile willughbiella*	359.43	95608	1.63	1000	1889.6	266	1
61	Lepidoptera	*Melanargia galathea*	173.41	7907	2.66	100	366.4	45.6	2
62	Diptera	*Melanostoma mellinum*	33.93	1439	3.57	200	477.9	42.4	5
63	Lepidoptera	*Melitae britomartis*	99.21	9475	3.51	100	1015.2	95.5	2

64	Hymenoptera	*Melitta* sp.	63.74	4290	3.25	100	876.8	67.3	2
65	Hymenoptera	*Melitturga* sp.	230.56	78275	2.03	750	2880.9	339.5	1
66	Diptera	*Minettia fasciata*	28.33	1708	6.4	100	1690.2	60.3	2
67	Lepidoptera	*Mompha* sp.	68.57	2585	2.95	100	614	37.7	2
68	Diptera	*Musca domestica*	77.72	9544	3.86	100	2070.3	122.8	2
69	Hymenoptera	*Nomada succincta*	136.27	20904	2.44	100	1442.6	153.4	2
70	Diptera	*Orellia falcata*	55.44	3199	3.44	100	759.5	57.7	2
71	Lepidoptera	*Ostrinia nubilalis*	34.32	2207	4.14	100	1291.1	64.3	1
72	Hymenoptera	*Panurgus calcaratus*	42.34	207	4.38	100	83.4	4.9	1
73	Diptera	*Paragus haemorrhous*	52.11	1225	3.33	100	571.9	23.5	1
74	Lepidoptera	*Pieris rapae*	163.19	6772	2.71	100	317.2	41.5	6
75	Diptera	*Pipizella viduata*	61.82	2133	3.28	100	992	34.5	1
76	Diptera	*Platycheirus clypeatus*	90.56	2454	2.68	100	618.4	27.1	14
77	Diptera	*Platypalpus pallidiventris*	8.93	158	7.77	100	277.4	17.7	1
78	Lepidoptera	*Plebeius idas*	94	6091	3.58	100	676.9	64.8	2
79	Diptera	*Pollenia pediculata*	85	7752	3.69	100	1471	91.2	3
80	Diptera	*Pollenia similis*	46.73	4323	4.98	100	2009.4	92.5	1
81	Lepidoptera	*Polyommatus agestis*	88.71	4906	3.64	200	734.1	55.3	2
82	Lepidoptera	*Polyommatus icarus*	99.87	9198	3.44	200	912.3	92.1	18
83	Hymenoptera	*Psenulus fuscipennis*	70.79	7497	3.94	100	1864.1	105.9	1
84	Lepidoptera	*Pyralidae sp.*	48.24	1650	3.49	100	579.4	34.2	1
85	Diptera	*Rhingia campestris*	278.35	44118	1.54	200	2147.4	158.5	1
86	Lepidoptera	*Rhodostrophia calabra*	57.84	1024	3.12	100	143.8	17.7	2
87	Diptera	*Sapromyza quadripunctata*	21.11	2727	4.38	100	1874.9	129.2	1
88	Diptera	*Sarcophaga haemorrhoa*	131.4	31155	3.24	200	4852.2	237.1	2
89	Diptera	*Sarcophaga* (s.str.) *carnaria*	287.03	26235	2.08	200	1247.9	91.4	2
90	Diptera	*Sarcophaga arcipes*	124.91	12878	3.31	150	2148	103.1	5
91	Diptera	*Sarcophaga lehmanni*	196.05	31564	2.65	200	2738	161	2
92	Diptera	*Sarcophaga variegata*	243.29	25399	2.38	150	1573.2	104.4	2
93	Diptera	*Scaeva pyrastri*	220.05	3565	1.98	100	222.8	16.2	1
94	Lepidoptera	*Scolitantides baton*	91.4	7687	3.66	200	931.1	84.1	1
95	Hymenoptera	*Selandria serva*	31.42	2756	4.89	100	1861.8	87.7	1
96	Diptera	*Shaerophoria interrupta* group	60.4	429	4.68	100	178.7	7.1	1

97	Diptera	*Siphona rossica*	24.99	1834	6.81	200	2184.2	73.4	1
98	Diptera	*Soleria pacifica*	51.14	13030	4.64	100	6548.5	254.8	1
99	Diptera	*Solieria vacua*	62.5	8044	4.2	100	2990.7	128.7	1
100	Diptera	*Spallanzania hebes*	256.17	24182	2.32	200	1383.8	94.4	1
101	Diptera	*Spallanzania multisetosa*	206.02	18006	2.59	200	1429.7	87.4	1
102	Diptera	*Sphaerophoria scripta*	65.58	3056	4.37	150	1199.6	46.6	23
103	Hymenoptera	*Sphecidae* sp.	112.57	24934	3.12	100	3093.5	221.5	1
104	Diptera	*Syritta pipiens*	55.98	2446	5.27	100	918.4	43.7	5
105	Diptera	*Syrphidae*	105.56	2248	2.62	100	282.8	21.3	1
106	Diptera	*Syrphus vitripennis*	107.5	9611	2.6	100	1175.7	89.4	1
107	Diptera	*Tachina fera*	306.39	16576	1.47	200	698.6	54.1	1
108	Hymenoptera	*Tenthredo notha*	154.17	4193	2.67	100	324.1	27.2	1
109	Hymenoptera	*Tetralonia* sp.	120.31	24146	2.37	500	1904.2	200.7	1
110	Lepidoptera	*Thymelicus sylvestris*	139.1	7386	2.94	100	440.7	53.1	2
111	Diptera	*Triglyphus primus*	24.61	458	5.19	100	846.6	18.6	1
112	Hymenoptera	*Vespula vulgaris*	140	18928	2.8	200	1692.5	135.2	1
113	Lepidoptera	*Zygena filipendulae*	188.64	7244	2.23	100	284.6	38.4	2
